# Trends in Physician Exit From Fee-for-Service Medicare

**DOI:** 10.1001/jamahealthforum.2025.2267

**Published:** 2025-07-18

**Authors:** Hannah T. Neprash, Michael E. Chernew

**Affiliations:** 1 Division of Health Policy and Management, School of Public Health, University of Minnesota, Minneapolis; 2 Department of Health Care Policy, Harvard Medical School, Boston, Massachusetts

## Abstract

This cohort study analyzed Medicare claims data between 2010 and 2024 to quantify the rate of physician exits from traditional Medicare.

## Introduction

Adequacy of the US physician workforce remains a perennial policy topic. Many observers are concerned that physicians will increasingly leave medicine. Existing evidence suggests that physician exit from Medicare increased through 2014 but plateaued through 2016.^1^ We analyzed Medicare data to quantify the post–COVID-19 pandemic rate of physician exit from traditional Medicare, as inflation-adjusted fees decrease and administrative and new clinical burdens increase.

## Methods

Using National Provider Identifiers (NPIs) and 100% of fee-for-service claims, we analyzed the annual number of Part B claims billed by each clinician to Medicare from 2010 to 2024. The University of Minnesota Institutional Review Board approved this cohort study and deemed it not research involving human participants and therefore exempt from informed consent. We followed the STROBE reporting guideline.

Using the Medicare Data on Provider Practice and Specialty files, we limited our sample to physicians and identified each physician’s broad specialty category (eg, primary care). We also calculated group practice size for each physician as the unique number of physician-NPIs billing within their Taxpayer Identification Number.^2^ To avoid conflating exit and intermittent Medicare billing patterns, we excluded physicians who, on average, billed fewer than 100 Medicare claims annually.

We defined exit as the absence of any Medicare claims for 12 consecutive months. With this lookback period and data through 2024, the last observed exits occurred in 2023. To adjust for physician age, we regressed an annual physician-level indicator for exit on calendar year and physician age. Stratified analyses included interaction of calendar year with physician specialty and group size. Analyses were conducted using Stata, version 18.0 (StataCorp).

## Results

**Figure 1.**
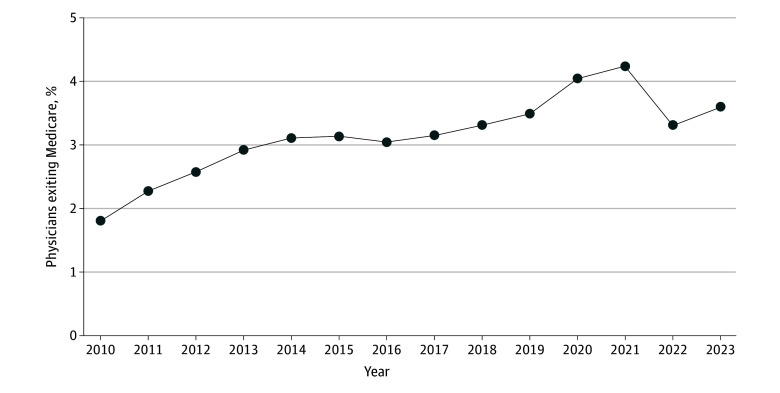
Age-Adjusted Annual Rate of Physician Exit From Medicare From 2010 to 2023

The sample included 791 025 physicians (mean [SD] age, 44.6 [12.0] years) billing Medicare between 2010 and 2024. The share of physicians exiting Medicare in any given year increased significantly from 1.80% (95% CI, 1.75%-1.85%) to 3.60% (95% CI, 3.56%-3.65%) (Figure 1). Physician exit displayed a gradual increase (2010-2013), a period of stability (2014-2016), a gradual increase (2017-2019), a spike during the pandemic (2020-2021), and a return in 2023 to levels above the 2019 rate.

**Figure 2.**
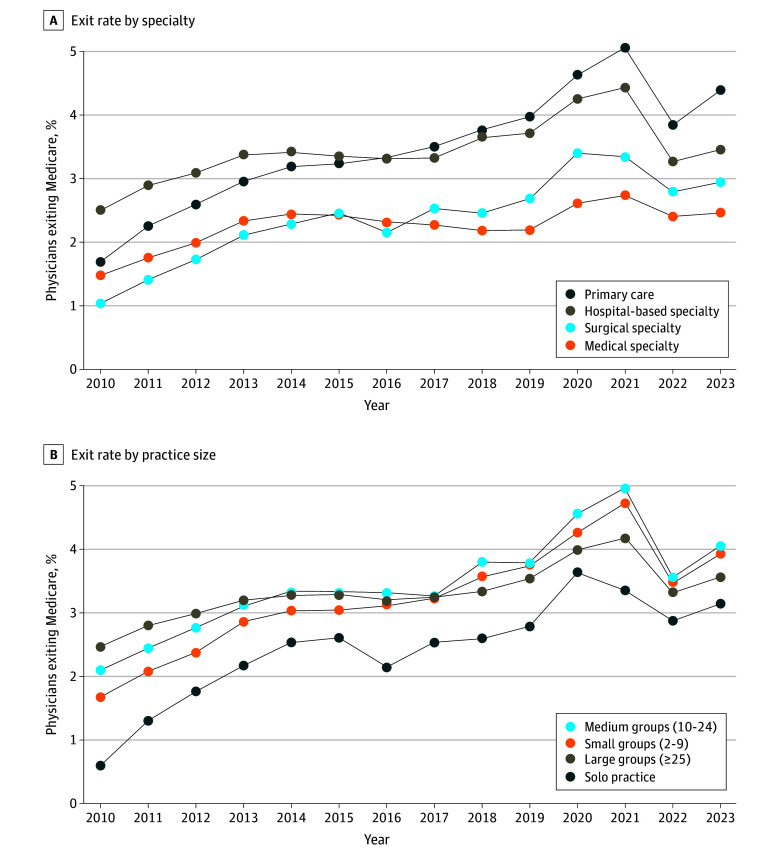
Age-Adjusted Annual Rate of Physician Exit From Medicare by Specialty and Group Practice Size From 2010 to 2023

In 2023, the exit rate among primary care physicians (PCPs; 4.41%; 95% CI, 4.33%-4.50%) exceeded that of hospital-based specialists (3.50%; 95% CI, 3.42%-3.58%), surgical specialists (2.99%; 95% CI, 2.88%-3.10%), and medical specialists (2.49%; 95% CI, 2.40%-2.59%) (Figure 2). From 2010 to 2023, the age-adjusted share of PCPs exiting Medicare increased by 0.21 (95% CI, 0.20-0.21) percentage points, which significantly exceeded annual growth in exit among surgical specialists (0.14 [95% CI, 0.14-0.15] percentage points), hospital-based specialists (0.08 [95% CI, 0.07-0.09] percentage points), and medical specialists (0.06 [95% CI, 0.05-0.06] percentage points). While age-adjusted exit rates in 2023 were lowest among physicians in solo practice (3.16%; 95% CI, 3.03%-3.30%), annual growth in exit among solo practitioners (0.18 [95% CI, 0.17-0.18] percentage points) exceeded that of physicians in medium (0.15 [95% CI, 0.14-0.16] percentage points) and large group practices (0.08 [95% CI, 0.08-0.09] percentage points).

## Discussion

Over time, physician exit from traditional Medicare has increased. This result is consistent with earlier findings,^1,3^ but exits remained high even after the pandemic, which likely accelerated some physicians’ exit. The findings may reflect multiple factors, including the greater burden of new communication methods (eg, portal messages) and demands for clinical documentation.^4^ More rapid growth in exit among small practices likely contributes to consolidated physician markets, given that new physicians increasingly work for large practices.^2^ Decreased fees may also play a role but cannot explain the 2014 to 2016 stabilization in exit rates. Variation in exit rates by specialty suggests that concern about inadequate PCP supply may be warranted but requires investigation.

Study limitations include a reliance on Medicare fee-for-service claims and inability to distinguish exit from Medicare vs exit from clinical practice or retirement. Additionally, we did not fully describe the overall physician supply since we did not include physician entry.

## Notes

Supplement.
Data Sharing Statement
