# Supplementary material for: Trends in Physician Exit From Fee-for-Service Medicare
Source: JAMA Health Forum. 2025 Jul 18;6(7):e252267. doi: 10.1001/jamahealthforum.2025.2267 (PMC12274972; doi:10.1001/jamahealthforum.2025.2267)
Supplement: Supplement. — Data Sharing Statement [file jamahealthforum-e252267-s001.pdf]

Data Sharing Statement

Neprash. Trends in Physician Exit From Fee-for-Service Medicare. *JAMA Health Forum*. Published July 18, 2025. doi:10.1001/jamahealthforum.2025.2267

Data
Data available: No

Additional Information
Explanation for why data not available: Data are governed by a Data Use Agreement with CMS.
